# Psychosocial predictors of breast self-examination behavior among female students: an application of the health belief model using logistic regression

**DOI:** 10.1186/s12889-017-4880-9

**Published:** 2017-11-03

**Authors:** Alireza Didarloo, Bahram Nabilou, Hamid Reza Khalkhali

**Affiliations:** 1 Social Determinants of Health Research Center, Department of Public Health, School of Health Sciences, Urmia University of Medical Sciences, P. O. Box: 57561-15111, Urmia, Iran; 2 Department of Public Health, School of Health Sciences, Urmia University of Medical Sciences, P. O. Box: 57561-15111, Urmia, Iran; 3 Patient Safety Research Center, Department of Biostatistics and Epidemiology, Faculty of Medicine, Urmia University of Medical Sciences, P. O. Box: 57561-15111, Urmia, Iran

**Keywords:** Breast cancer, Breast self-examination, Health belief model, Student, University

## Abstract

**Background:**

Breast cancer is a life-threatening condition affecting women around the world. The early detection of breast lumps using a breast self-examination (BSE) is important for the prevention and control of this disease. The aim of this study was to examine BSE behavior and its predictive factors among female university students using the Health Belief Model (HBM).

**Methods:**

This investigation was a cross-sectional survey carried out with 334 female students at Urmia University of Medical Sciences in the northwest of Iran. To collect the necessary data, researchers applied a valid and reliable three-part questionnaire. The data were analyzed using descriptive statistics and a chi-square test, in addition to multivariate logistic regression statistics in SPSS software version 16.0 (SPSS Inc., Chicago, IL, USA).

**Results:**

The results indicated that 82 of the 334 participants (24.6%) reported practicing BSEs. Multivariate logistic regression analyses showed that high perceived severity [OR = 2.38, 95% CI = (1.02–5.54)], high perceived benefits [OR = 1.94, 95% CI = (1.09–3.46)], and high perceived self-efficacy [OR = 13.15, 95% CI = (3.64–47.51)] were better predictors of BSE behavior (*P* < 0.05) than low perceived severity, benefits, and self-efficacy. The findings also showed that a high level of knowledge compared to a low level of knowledge [OR = 5.51, 95% CI = (1.79–16.86)] and academic undergraduate and graduate degrees compared to doctoral degrees [OR = 2.90, 95% CI = (1.42–5.92)] of the participants were predictors of BSE performance (*P* < 0.05).

**Conclusions:**

The study revealed that the HBM constructs are able to predict BSE behavior. Among these constructs, self-efficacy was the most important predictor of the behavior. Interventions based on the constructs of perceived self-efficacy, benefits, and severity are recommended for increasing women’s regular screening for breast cancer.

## Background

Cancer, a dangerous and fatal disease, is a public health challenge in most populations of the world. In 2012, over fourteen million new cases of cancer and 8.2 million cancer-related deaths occurred worldwide. Fifty-seven percent of the new cases and 65 % of the cancer-related deaths occurred in less developed countries [1]. Among the Iranian population, cancer is the third leading cause of death after heart disease and accidents. It has also been estimated that cancer incidence rates will reach 98 to 100 per 100,000 individuals annually [2]. Breast cancer accounts for 23% of all cancers in women, and as a chronic condition, it is the second leading cause of cancer-related deaths in women around the world [1, 2]. The global rate of breast cancer incidence ranges from 27 per 100,000 individuals in African and Middle Eastern countries to 96 per 100,000 individuals in Western European countries [1]. Breast cancer is the most common cancer among Iranian women, with an incidence rate of 25 per 100,000 individuals [3].

With regard to the increasing age and life expectancy of the Iranian population, it is predicted that the number of breast cancer cases will increase in the coming years. In addition, each year, more than 502,000 Iranian women will die due to this cancer [4]. Previous evidence indicates that this cancer affects Iranian women at least ten years younger than women in developed countries, with the mean age of diagnosis between 47.1 and 48.8 years [5].

Early detection can accelerate the process of cancer treatment, significantly reduce mortality, and improve women’s overall quality of life. Some studies suggest that the early detection of breast cancer through screening tests can decrease mortality rates by 25–30% [6]. The National Breast Cancer Screening Program (NBCSP) introduced in 2012 recommends the following plan for breast cancer screening: (1) mammography should be conducted in women over the age of 40 annually, (2) a clinical breast examination (CBE) should be performed on women aged 20 to 40 years once every three years and on women over age 40 once a year, and (3) a breast self-examination (BSE) should be performed monthly by women over the age of 20 [7].

Despite the potential benefits of screening in detecting breast cancer, many women still do not undergo the recommended screening tests [8]. Understanding and improving factors related to women’s behavior regarding breast cancer screening tests will be beneficial in preventing breast cancer. Identifying these factors also helps researchers to design and implement appropriate interventions in behavioral change [9]. Furthermore, behavioral change interventions are likely to be more effective when they focus on theory-centered determinants [10, 11]. For instance, findings from a study performed in Iran by Tavafian et al. indicated that the perceived benefits and perceived self-efficacy of the women who performed BSEs were significantly higher than those who did not practice BSEs. Furthermore, perceived barriers were lower among those who had performed BSEs [12]. In their research using two behavior change models [Health Belief Model (HBM) and the Transtheoretical Model (TTM)] in South Korea, Kung Hur et al. found that perceived benefits, perceived susceptibility, and perceived barriers were the principal predictors of undergoing mammography screening [13].

The Health Belief Model (HBM) is one of the most important behavior change models and has been widely used to examine beliefs related to preventive health behaviors such as BSE, CBE, and mammography [14]. The HBM was developed by Becker (1974) [15] and modified by Rostenstock (1990) [16], and it may be beneficial in exploring the factors influencing women’s breast cancer screening behaviors [17].

The HBM includes six constructs: (1) perceived susceptibility shows a person’s perception towards the risk of a specific disease; (2) perceived seriousness determines a person’s feeling towards the side effects of a disease; (3) perceived benefits indicates a person’s perception towards the positive health consequences of performing a specific health behavior; (4) perceived barriers highlight a person’s perception of the costs or barriers to a given health behavior; (5) health motivation refers to an individual’s beliefs and behaviors towards a general health concern; and (6) self-efficacy refers to a person’s confidence in his/her ability to adopt a given health behavior. According to this model, when a woman feels that she is vulnerable to a specific disease or when she perceives that the disease has reached a dangerous state, she will be more likely to perform breast cancer-related screening behaviors [18]. Among breast cancer screening tests, the BSE is still considered a simple, noninvasive, inexpensive, affordable and accessible method for women who are younger and at a high risk to detect early changes in their breasts [19]. Most studies regarding breast cancer in Iran have been conducted on older women. This study is the first research performed on younger Iranian females, specifically students. This study applied the HBM as a theoretical basis to explore variables affecting BSE behavior among a sample of female university students in Urmia in Northwest Iran.

## Methods

### Design

This cross-sectional descriptive study was carried out with a sample of female students at Urmia University of Medical Sciences in Northwest Iran to assess predictors of BSE behavior. A convenience sample consisting of 334 eligible female university students was recruited and assessed from December 2014 to February 2015. The quota sampling method, which is a non-probability approach, was used for the sampling of the data. The inclusion criteria were as follows: 1. female student, 2. studying at Urmia University of Medical Sciences as an undergraduate/graduate medical, dentistry, or pharmaceutical student, 3. between 20 and 30 years of age, and 4. willing to participate in the study. The sample size was comparable to what other researchers have previously used in the professional literature [20]. Considering the fact that performed BSE behavior by female students in the study conducted by Akhtari-Zavare et al. [21] was 26%, we used a 95% confidence interval (CI) and an accepted 5% stated absolute error and calculated the sample size using the following formula:

$$ \mathrm{Sample}\  \mathrm{size}=\mathrm{z}{2}^{\ast }{\mathrm{p}}^{\ast}\mathrm{q}/\mathrm{d}2 $$

$$ \mathrm{Z}=1.96\ \mathrm{for}\ 95\%\mathrm{CI}. $$

$$ \mathrm{p}=\mathrm{proportion}\  \mathrm{of}\ \mathrm{BSE}. $$

$$ \mathrm{q}=1-\mathrm{p}. $$

$$ \mathrm{d}=\mathrm{absolute}\  \mathrm{error}/\mathrm{accepted}\  \mathrm{value}. $$

To address the effect of subject attrition, we increased our sample size by 20%.

### Instrument

To collect the study data, researchers developed a 20-min self-report questionnaire using previous scientific literature and scales. This instrument included the following three subscales:

1. Demographic information that examined variables such as participants’ academic year in school, the grade point average, academic level, age, ethnicity, frequency of performing breast self-examinations, and number of first- and second-degree relatives affected by breast cancer.
2. The Breast Cancer Knowledge Test (BCKT) developed by McCance et al. (1990) was applied to measure participants’ knowledge. The BCKT is a 19-item instrument that measures subjects’ knowledge of breast cancer detection and screening practices. Each correct response was scored as 1, and each false and “do not know” response was scored as 0.
3. The Champion’s Revised Health Belief Model Scale (CRHBMS) (1999) was used to measure participants’ health beliefs towards breast cancer and its screening tests. The CRHBMS encompasses 41 items and utilizes a 5-point Likert scale ranging from 1, “strongly disagree”, to 5, “strongly agree”. The CRHBMS comprises eight subscales: five items related to susceptibility, seven items related to seriousness, six items related to the benefits of the BSE, five items related to barriers to BSE, eleven items related to self-efficacy (confidence), and seven items related to health motivation.

To determine the status of participants’ knowledge on each of the model constructs, the total obtained scores were categorized into three groups: the first one-third had low status; the second one-third to two-thirds had moderate status; and the last one-third had high status.

The multi-part questionnaire used in the present study was in English originally. It was translated into the Persian language and then underwent the back-translation technique. Furthermore, to determine the validity of the translated questionnaire, the researchers utilized a qualitative method of content validity, specifically a panel of experts. First, the study instrument was sent via email to 10 academics who acted as the panel of experts and examined the items in terms of necessity, relevance, and clarity. Then, feedback received from the specialists was analyzed, and suggested changes were made regarding the study instrument. Finally, the content validity of the finalized questionnaire was confirmed.

To determine the reliability of the study questionnaire, it was completed by all the participants. Next, the Cronbach’s alpha coefficient was computed for the BCKT instrument, the CRHBMS questionnaire, and its subscales. The alpha value for the BCKT and CRHBMS questionnaires were 0.75.2 and 0.82.2, respectively. In addition, the alpha value of the CRHBMS subscales for the current study ranged from 0.75 to 90 (Table 1). Therefore, the reliability of the study instruments was also confirmed.

**Table 1:** Subscale scores of CRHBMS^a^ regarding breast cancer and breast self-examination

Subscale	No. of items	Cronbach’s α	Mean	SD^b^
Perceived susceptibility	5	0.749	2.64	0.61
Perceived severity	7	0.797	2.95	0.75
Perceived benefits	6	0.846	3.51	0.67
Perceived barriers	5	0.880	2.10	0.85
Health motivation	7	0.766	3.65	0.66
Perceived self-efficacy	11	0.897	2.85	0.75

^a^CRHBMS = Champion’s Revised Health Belief Model scale; ^b^SD = Standard Deviation

### Statistical analysis

To analyze the study data, the researchers applied descriptive and inferential statistical methods. A chi-square test was conducted to assess the relationship between two categorical variables. In addition, a multivariate logistic regression was applied to determine the factors affecting behavior using SPSS software version 16.0 (SPSS Inc., Chicago, IL, USA). In this study, a *P* value less than 0.05 was considered significant in all the analyses.

## Results

### Demographics and BSE behavior

In total, 366 eligible subjects were recruited into the study and were asked to fill out the questionnaire. Thirty-two questionnaires were excluded due to either the participant not following the instructions provided by the researchers (20 cases) or incompleteness (12 cases). Hence, statistical analyses were performed on 334 questionnaires.

The obtained results indicated that 82 out of 334 participants (24.6%) performed BSEs. To determine the probable predictors of BSE, demographic factors were compared between the groups that performed and did not perform BSE behavior. In the group with BSE behavior, 85.4% of the participants were undergraduate/graduate students, and in the group without BSE behavior, 66.3% were undergraduate/graduate students. Academic level had a statistically significant association with BSE behavior (*P* < 0.001, Table 2).

**Table 2:** Association of the demographic and HBM factors with Breast-Self-Examination behavior

Independent Continuous variable	BSE performance				*P*-value
	No		Yes		
	Mean	SD	Mean	SD	
Age	21.75	1.61	22.10	1.72	0.1
Independent categorical variables	Count	Percentage	Count	Percentage	
Academic level:					
General Doctor	85	33.7	12	14.6	0.001
Undergraduate/Graduate	167	66.3	70	85.4	
Academic year					
First	74	29.4	20	24.4	0.045
Second	56	22.2	22	26.8	
Third	96	38.1	23	28.1	
Fourth and higher	26	10.3	17	20.7	
Ethnicity					
Turk	150	59.5	51	62.2	0.892
Kurd	84	33.3	25	30.5	
Others	18	7.1	6	7.3	
The grade point average					
Low (<14)	18	7.1	3	3.7	0.028
Moderate (14–15.99)	110	43.7	24	29.3	
Good (16–17)	92	36.5	37	45.1	
Excellent (>17)	32	12.7	18	22	
Family history of breast cancer					
No	224	88.9	78	95.1	0.096
Yes	28	11.1	4	4.9	
Participant s’ knowledge					
Low	69	27.4	5	6.1	<0.001
Moderate	143	56.7	31	37.8	
High	40	15.9	46	56.1	
Perceived self-efficacy					
Low	79	31.3	6	7.3	<0.001
Moderate	166	65.9	62	75.6	
High	7	2.8	14	17.1	
Perceived benefits					
Low/Moderate	144	57.1	29	35.4	0.001
High	108	42.9	53	64.6	
Perceived barriers					
Low	177	70.2	63	76.8	0.249
Moderate/High	75	29.8	19	23.2	
Perceived severity					
Low	37	14.7	9	10.9	0.008
Moderate	158	62.7	40	48.8	
High	57	22.6	33	40.3	
Perceived susceptibility					
Low	68	27	20	24.4	0.643
Moderate/High	184	73	62	75.6	
Health motivation					
Low	9	3.6	2	2.4	0.283
Moderate	86	34.1	23	28	
High	157	62.3	51	69.6	

Academic year showed a significant association with BSE behavior. We found that 20.7% of the participants in the group with BSE behavior and 10.3% in the group without BSE behavior were in the fourth year and higher (*P* = 0.045, Table 2). Approximately 62.2% of the subjects in the group with BSE behavior and 59.5% of participants in the group without BSE behavior were of Turkish ethnicity (*P* = 0.892, Table 2). A significant association was observed between the grade point average of the participants and their BSE behavior. In the group with BSE behavior, over 67.1% of the participants had good or excellent grade point averages, and in the group without BSE behavior, 49.2% of the participants had good or excellent grade point averages (*P* = 0.028, Table 2).

The findings also showed that 4.9% of the participants in the group with BSE behavior and 11.1% of the participants in the group without BSE behavior had a family history of breast cancer. However, no significant relationship was seen between participants’ family history of breast cancer and their BSE behavior (*P* = 0.096, Table 2). The study highlighted that 56.1% of the students belonging to the group with BSE behavior had a high level of knowledge. Moreover, 15.9% of the other group reported having a high level of knowledge as well. Statistical analyses showed that a significant relationship existed between students’ knowledge and their BSE behavior (*P* < 0.001, Table 2).

### HBM constructs and BSE behavior

In this research, we also assessed the association between BSE behavior and the HBM constructs.

The study findings indicated that 17.1% of the group with BSE behavior and 2.8% of the other group had high perceived self-efficacy towards BSE. A statistically significant association was also observed between participants’ self-efficacy and their BSE behavior (*P* < 0.001, Table 2). Approximately 64.6% of the participants in the group with BSE behavior and 42.9% of the other group perceived the behavior as highly beneficial. The association between perceived benefits and BSE behavior was statistically significant (*P* = 0.001, Table 2).

According to the study results, 23.2% of the students belonging to the group with BSE behavior and 29.8% of the group members without BSE behavior perceived moderate/high barriers to adopting BSE behavior. Statistically, there was no significant relationship between the participants’ perceived barriers and their BSE behavior (*P* = 0.249, Table 2).

Moreover, approximately 40.3% of the students belonging to the group with BSE behavior and 22.6% of the students without BSE behavior reported a high perceived severity of BSE behavior. The association between perceived severity and BSE behavior was statistically significant (*P* = 0.008, Table 2).

To understand the real factors associated with BSE behavior, the significant variables in the univariate analysis were inserted and tested in a multivariate logistic regression model using the forward method. The regression results showed that, compared to the respondents with a low level of knowledge, those with a moderate level [OR = 1.73, 95% CI = (0.61–4.92), Table 3] and high level of knowledge [OR = 5.51, 95% CI = (1.79–16.86), Table 3] were more likely to practice BSE. The odds ratio for respondents in undergraduate/graduate levels compared to general doctoral students who performed BSE behavior was 2.90, 95% CI = (1.42–5.92), Table 3. The students with moderate perceived severity [OR = 1.04, 95% CI = (0.46–2.33), Table 3] and high perceived severity [OR = 2.38, 95% CI = (1.02–5.54), Table 3] were more likely to perform BSEs than participants with low perceived severity. Participants who had high perceived benefits of BSE [OR = 1.94, 95% CI = (1.09–3.46), Table 3] were more likely to practice BSEs than participants with low/moderate perceived benefits.

**Table 3:** Factors associated with breast self-examination based on multivariate logistic regression model

Independent variables	OR (95% CI)	*P*-value
Academic level		.003
Professional Doctor	Reference	NA
Undergraduate/Graduate	2.90 (1.42–5.92)	.003
Participants’ knowledge		.001
Low	Reference	NA
Moderate	1.73 (.61–4.92)	.302
High	5.51(1.79–16.86)	.003
Perceived self-efficacy towards breast self-examination		<0.001
Low	Reference	NA
Moderate	4.82(1.95–11.89)	.001
High	13.15(3.64–47.51)	<0.001
Perceived benefits towards breast self-examination		0.024
Low/Moderate	Reference	NA
High	1.94(1.09–3.46)	0.024
Perceived severity towards breast cancer		0.039
Low	Reference	NA
Moderate	1.043(0.46–2.33)	0.923
High	2.38(1.02–5.54)	0.044

CI = confidence interval; NA = not applicable; OR = odds ratio

Finally, the odds ratios for respondents with moderate and high perceived self-efficacy compared to students with low perceived self-efficacy in performing BSEs were 4.82, 95% CI = (1.95–11.89), Tables 3 and 13.15, 95% CI = (3.64–47.51), Table 3, respectively.

Overall, the study model had a strong goodness of fit and explained 64% of the variance of BSE behavior, and participants’ perceived self-efficacy was found to be the most important predictor of the behavior among the HBM variables.

## Discussion

The purpose of this research was to assess predictors of BSE behavior based on the HBM among female students at Urmia University of Medical Sciences in Northwest Iran. The BSE is one screening method for identifying breast cancer in women who live in regions of the world with no access to advanced screening tests [22]. Correct and regular performance of BSEs can help women detect unusual changes in their breasts related to breast cancer. It has been reported that early detection plays a role in the prompt treatment of cancer and yields a better survival rate [23].

In the current survey, only 24.6% of the respondents performed BSEs. This finding is supported by some studies such as one conducted by Akhtari-Zavare et al. in Malaysia that reported that only 26% of the participants performed BSEs [21]. A study conducted by Erbil et al. in Turkey indicated that only 21.8% of nursing students practiced BSEs every month [24]. The low frequency of BSEs in female university students can be due to different factors. A lack of knowledge about women’s health is a factor that can impede preventive practices against different diseases. In this study, the participants who had a high level of knowledge performed BSEs 5.51 times more than those with a low level of knowledge. It means that knowledge plays a principal role in adopting and conducting health promoting behaviors.

Previous studies also supported and confirmed this part of our findings. A study conducted by Canbulat and Uzun in Turkey indicated that knowledge of breast cancer (BC) and awareness of BC screening methods influenced BSE behavior [25]. A study by Elsie et al. in Uganda (2010) [26] and Okolie in Nigeria (2012) [27] also noted a significant relationship between participants’ overall knowledge regarding breast cancer and their frequency of BSE performance. Having adequate knowledge of the risk factors related to breast cancer is the foundation of primary prevention of breast cancer [28]. Students’ knowledge helps them detect breast cancer and accelerate its treatment. After teaching young females, they can then teach their mothers and siblings, too. In this way, breast cancer incidence may be reduced.

People’s knowledge cannot always protect them from diseases and other health problems. Knowledge is a necessary condition; however, it is not sufficient. In addition to knowledge, individuals’ beliefs towards health issues and their preventive behaviors play the main role in facilitating or impeding health promoting behaviors [29]. Regarding health attitudes, Burnett et al. suggested that people’s attitudes towards health behaviors must be strengthened [30].

This study found that the participants who believed in the benefits of BSE behavior performed the behavior 1.94 times more than the other groups. The results of a study done by Wardle et al. in Europe and Tastan et al. in Turkey were consistent with our study findings. They reported that an individual’s attitudes towards the benefits of BSE are related to performing BSEs [20, 31]. Graham (2002) also stated that among women, those who perceived a benefit from BSE had higher odds of performing the screening than those who did not see the screening as beneficial [32]. Furthermore, a survey by Shiryazdi et al. in Iran revealed that female health workers who performed BSE and mammography practices had significantly higher perceived benefits than those who did not engage in these behaviors [33]. With regard to the above results, we concluded that perceived benefits play an important role in adopting preventive behaviors such as BSE behavior.

In addition, the results revealed that the participants with high perceived self-efficacy were 13.15 times more likely to perform BSE than those with lower perceived self-efficacy. The studies conducted in Iran by Masoudiyekta et al. and Hasani et al. indicated that perceived self-efficacy was the most powerful predictor of BSE [34, 35]. Another study conducted in the United States also clarified that perceived self-efficacy was a predictor of performing BSEs among American women [36]. Moreover, Jirojwong and MacLennan found that self-efficacy (confidence) was one of the important determinants of regular BSE [37].

The findings of the above studies were consistent with this part of our findings, specifically that self-efficacy levels can increase or impede the motivation to act. People with high self-efficacy choose to engage in more challenging tasks. In performing a preventive health practice, highly self-efficacious persons invest more effort and persist longer than those with low self-efficacy. It is therefore recommended that educational interventions be designed and carried out to enhance the skills and efficacy of young women regarding self-examinations of their breasts.

Based on the findings of the present research, students’ perceived severity, as part of perceived threat, was statistically correlated with their BSE performance. Those with a high perceived severity of breast cancer performed the BSE behavior 2.38 times more than those with low perceived severity. A study by Jirojwong and MacLennan in Australia revealed that perceived threat due to breast cancer is an important factor that influences BSEs among Thai migrant women [37].

In a study by Savage and Clarke, a relationship between the belief of susceptibility (as part of perceived threat) to breast cancer and BSE was found among Australia-born women in Australia [38]. These results are consistent with the findings of the present study. Health care providers can provide information about risk factors for breast cancer to increase women’s perceived sensitivity and severity related to the cancer.

Finally, our study found a strong association between academic level and adherence to BSE among the studied students. Participants in undergraduate and graduate levels performed BSEs 2.90 times more than other students. A possible reason for this finding could be that students with undergraduate and graduate degrees paid more attention to the practices of primary prevention than the medical group working in clinical areas.

This study, similar to other research studies, has a number of limitations. First, this investigation is a cross-sectional study that can only determine associations between variables, and it is not capable of examining cause and effect relationships between the variables. Second, the results of this study can be generalized only to similar samples and not beyond. Finally, the study data were collected via a self-reported questionnaire. Thus, participants may have underestimated or overestimated their BSE behavior, which in turn might have affected the study findings.

## Conclusions

This study highlighted that breast self-examination behavior among female university students was low and far from favorable levels. The study documented that participants’ knowledge, perceived benefits, self-efficacy, perceived severity, and academic level were factors that influence their decisions related to performing BSEs. Of the CRHBMS subscales, perceived self-efficacy was the most important construct that explained BSE. It is recommended that health personnel consider the above variables when designing educational interventions regarding breast cancer and its screening tests. Further research is recommended using a larger sample size from similar populations. These studies can be helpful in identifying factors that influence the use of BSE.
